# Local social environmental factors are associated with household food insecurity in a longitudinal study of children

**DOI:** 10.1186/1471-2458-12-1038

**Published:** 2012-11-28

**Authors:** Megan Ann Carter, Lise Dubois, Mark S Tremblay, Monica Taljaard

**Affiliations:** 1 Institute of Population Health, University of Ottawa, 1 Stewart St, Ottawa (ON), Canada, K1N 6N5; 2 Department of Epidemiology and Community Medicine, University of Ottawa, 451 Smyth Road, Ottawa (ON), Canada, K1H 8M5; 3 Healthy Active Living and Obesity Research, Children’s Hospital of Eastern Ontario Research Institute, 401 Smyth Road, Ottawa (ON), Canada, K1H 8L1; 4 Ottawa Hospital Research Institute, Clinical Epidemiology Program, 725 Parkdale Avenue, Ottawa (ON), Canada, K1Y 4E9

**Keywords:** Food insecurity, Social capital, Social cohesion, Disorder, Deprivation, Neighbourhood, Longitudinal study, Environment, Context

## Abstract

**Background:**

Food insecurity is a significant public health problem in North America and elsewhere. The prevalence of food insecurity varies by country of residence; within countries, it is strongly associated with household socioeconomic status, but the local environment may also play an important role. In this study, we analyzed secondary data from a population-based survey conducted in Québec, Canada, to determine if five local environmental factors: material and social deprivation, social cohesion, disorder, and living location were associated with changes in household food insecurity over a period of 6 years, while adjusting for household socioeconomic status (SES) and other factors.

**Methods:**

Data from the Québec Longitudinal Study of Child Development, following same-aged children from 4–10 y of age, were analyzed using generalized estimating equations, to determine the longitudinal association between these environmental factors and food insecurity over a period of 6 years.

**Results:**

Of the 2120 children originally included in the cohort, 1746 (82%) were included in the present analysis. The prevalence of food insecurity was 9.2% when children were 4 y of age (95% CI: 7.8 – 10.6%) but no significant changes were observed over time. On average over the 6 year period, three environmental factors were positively related to food insecurity: high social deprivation (OR 1.62, 95%CI: 1.16 – 2.26), low social cohesion (OR 1.45 95%CI: 1.10 – 1.92), and high disorder (OR 1.76, 95%CI: 1.37 – 2.27), while living location and material deprivation were not related to food insecurity. These associations were independent of household SES and other social variables.

**Conclusion:**

These results highlight the potential role of the local social environment in preventing and ameliorating food insecurity at the household level. Stakeholders providing food security interventions at the community level should consider interactions with local social characteristics and perhaps changing the social environment itself. Further intervention research also examining interactions with household-level factors could lead to the development of interventions that increase both household and community-level food security.

## Background

In high income countries, health problems associated with positive caloric balance are common. What is less well known is that a portion of residents in these countries do not reap the benefits of prosperity; some residents do not always get enough to eat and are therefore considered food insecure. Food insecurity exists when there is limited or uncertain access to nutritionally adequate and safe foods or limited or uncertain ability to acquire acceptable foods in socially acceptable ways [1]. It is linked to lack of financial resources and is composed of several dimensions, including the quality and quantity of foods, anxiety about having enough to eat, and negative impacts on social interactions [2]. In 2007–2008, almost one million (7.7%) Canadian households were food insecure [3]. This is in contrast to the US, where 14.5% of American households in 2010 were food insecure [4].

Aside from being ethically unacceptable, the occurrence of food insecurity in developed countries has been linked to a number of health conditions, such as developmental problems in children [5,6], and depression among adults [7,8]. Other health correlates include obesity [9], cardiovascular disease risk factors [10], and delaying health care [11].

Food insecurity is strongly related to household income level [12-14], although not all households living in poverty are food insecure [14]. Children may be especially vulnerable as the prevalence of food insecurity tends to be higher in households with children compared to households without children [3,4]. Other individual-level factors found to relate to food insecurity after adjustment for income level include single-parent family status, minority-status [15], and smoking status of household members [16].

Individual-level attributes, however, do not tell the whole story. It is also well-known that macro socio-political factors play a key role in the existence of food insecurity. For instance, at a global level, the degree of human development of countries is tightly linked to food insecurity, hunger, and undernourishment [17]. Active public policies to decrease poverty are seen as major ways to ensure food security for all [13,15]; thus, even among developed countries, the prevalence of food insecurity varies [18]. One study has even found US state-level characteristics such as low average wages, high rental housing costs, and residential instability, to be significantly related to food insecurity [19].

Consistent with a social-ecological theory of health that considers multiple levels of influence, it is less clear whether more local levels of social organization could influence the food security status of local residents. Characteristics of the community food system such as availability of food stores, food availability in schools and workplaces, and local policies, coupled with individual and collective social factors, may affect food availability and accessibility for households living in a particular area [20]. Understanding how these factors relate to food insecurity could inform approaches to sustainable food system reforms that could help to combat this public health concern.

Of interest in this study are variables that capture aspects of the physical and social environments of local places. These include material disadvantage, social capital (including social cohesion and social deprivation), disorder, and living location. The following paragraphs describe how these may be relevant for promoting or preventing individual or household food insecurity.

Disadvantaged areas may have fewer healthy food services and resources, such as supermarkets or large grocery stores, compared to more affluent areas. The existence of these areas, often called food deserts, has been documented in the US, but is more controversial in other developed countries [21]. Even if disparities in food access do not exist, food stores available in disadvantaged areas may sell foods of lower quality [22], prices may be higher or lower depending on the type of food store and type of food, and discount shops or convenience stores selling unhealthy foods may be more accessible in more deprived areas [23,24].

In place-based research, social capital is thought of as a group-level attribute, but does not have a consistent definition [25]. In broad terms it identifies the richness of social connections, trust, shared norms, and reciprocity among residents living within an area [26]. High social capital may allow residents to obtain food from neighbours or other institutions more easily in times of need, and mobilize for collective action to address food insecurity issues. Additionally, neighbourhood disorder, sometimes resulting from a break-down in the social structure of the area, may dissuade food service establishments, and other institutional supports from locating in particular areas, and fear may prevent residents from accessing nearby food resources [27]. High disorder may itself also negatively impact social capital.

The degree of development in an area, in terms of living location or urban/rural status, may also help to explain food insecurity. Fewer services and resources are available in rural areas [23,28], and the quality of foods may vary depending on the location of stores [22]. Growth in supermarket size and food system innovations have enabled prices to decrease and quality to increase [29]. Because large tracks of land are needed, these stores are increasingly relocating from urban to suburban areas [29], which may impact negatively on food availability and access for disadvantaged urban dwellers.

The literature examining area disadvantage, measures of social capital, disorder or living location in relation to individual or household food insecurity is sparse and has been conducted, for the most part, in the US. In general, these studies have relied exclusively on cross-sectional data, with many concentrating on low income or ethnic subgroups, making it difficult to compare to the rest of the population. Among these studies, food insecurity has been variously measured. Examples include use of the 18-item United States Department of Agriculture (USDA) Food Security Survey Module (FSSM), the shorter 6-item FSSM version, different items from the Radimer/Cornell and Community Childhood Hunger Identification Project (CCHIP) instruments, and the US food insufficiency question.

Perhaps due to a focus on low income populations by many of these studies, few have examined material disadvantage specifically. None, to our knowledge, have examined neighbourhood disorder. Some studies examining measures of collective efficacy or other measures of neighbourhood social capital have found an inverse relationship between social capital and food insecurity [30-33], although, others have uncovered null results [34-37]. Associations for living location have been more consistent, with findings pointing towards a protective effect of rural living [19,36,38-42]. There have been, on the other hand, studies that have had opposite [31] or null findings [30,43,44].

More robust studies are needed that account for changes in variables over time, as are studies adjusting for potential confounding effects of area disadvantage. Associations may also be different in different country or community contexts (e.g. US versus other countries), and state/provincial/regional or nationally representative samples may reduce bias and increase power to detect significant differences compared to low-income, convenience samples drawn from particular counties or cities/towns.

The purpose of this study, therefore, was to analyze data from a representative longitudinal study of children living in the province of Québec (Canada), in order to determine if material deprivation, social deprivation, social cohesion, disorder, and living location were associated with changes in the prevalence of food insecurity over time. Hypotheses were that low social cohesion, high disorder, and high material/social deprivation would each increase the likelihood of food insecurity, even after accounting for household socioeconomic status (SES) and other potentially important explanatory factors. Even though some previous studies have found rural living to be negatively associated with food insecurity, it was hypothesized that rural living would be positively associated, given than another study conducted in Québec uncovered disparities in food access by location of residence, with less access for residents in rural areas [23].

## Methods

This study was a secondary data analysis based on the Québec Longitudinal Study of Child Development (QLCSD). The QLSCD is an ongoing cohort study conducted by the Provincial Government of Québec to determine important factors operating early in life that may influence the health and development of young children born and raised in Québec [45]. Cohort children have been participating since 1997–1998 when they were five months of age, and are representative of same aged children in the Québec population.

In the QLSCD, a stratified, three-stage random sampling strategy was used to select children for inclusion [45]. Strata were based on geography (regions and degree of remoteness) and on birthrate. The 1997–1998 Québec Birth Registry was used as the sampling frame. Sampling occurred throughout the year to minimize the effect of seasonality. Twins, children with major diseases at birth, and those living on remote Native lands and reserves were excluded [45].

The original sample at the first data collection cycle contained 2223 children, representing a response rate of 83%; 2120 children were retained for the longitudinal portion of the study [45]. Data were collected by trained interviewers every 12 months starting at approximately five months of age. By age four, timing of data collection changed to coincide with the school year and continued to occur once a year until age eight, after which it occurred every other year, in order to minimize respondent burden [46]. Data were largely collected through computer-assisted personal interviewing in the child’s home, although some information, like food insecurity, was captured via mother self-completed questionnaire [45]. To date, 14 cycles of data are available (five months to 13 y of age). The present study uses data primarily from four, eight, and 10 y of age.

The QLSCD received ethics approval from an ethics board of the Québec Provincial Government [47]. Approval for the present data analysis was given by the University of Ottawa Research Ethics Board.

### Dependent variable – food insecurity

Food insecurity was measured three times, when children were approximately four, eight, and 10 y of age. It was based on three questions, adapted from the Radimer/Cornell hunger and food insecurity measure, which the mother answered as part of a larger self-completed questionnaire [48]. It has been used in other province-wide surveys and has been validated as part of a previous study [49,50].

The first two questions of the measure assess food insecurity at the household level by focusing on its qualitative and quantitative aspects (“We eat the same things several days in a row because we only have a few different kinds of food on hand, and don’t have enough money to buy more”, and “We eat less than we should because we don’t have enough money for food”). The third question assesses the qualitative component of food insecurity at the child level (“We can’t provide balanced meals for our children because we can’t afford it financially”). Response options for all three questions include: “often true”, “sometimes true”, and “never true”. Children were considered to be living in a food insecure household if the mother answered “sometimes” or “often true” to any of the three questions. Categorizing food insecurity based on any affirmative response (sometimes or often) is consistent with previous research [51]. Responses were considered missing at any one data collection cycle if the mother did not answer any of the three questions.

### Explanatory variables of interest – place factors

Five variables measured in the QLSCD captured aspects of place that may be related to food insecurity. These are the main independent variables of interest in this study and are discussed in more detail below. All original variables were previously derived by the Institut de la statistique du Québec (ISQ) or affiliated statisticians. Raw area information, such as postal codes, was not available for analyses.

### Area-level deprivation

Pampalon and colleagues have developed an index to measure area-level deprivation, originally to aid in health and social services planning in Québec [52,53]. The index is largely based on the work of Peter Townsend. In the QLSCD, the ISQ collected postal codes from participating children at five months of age (first data collection cycle) and then linked these to 1996 census data describing enumeration areas. Using principal components analysis, six census indicators were used to derive the two main dimensions: material and social deprivation. The following paragraph describes the indicators making up each dimension that were derived in the first data collection cycle.

Using principal components analysis, material deprivation factor scores were calculated from the proportion of persons ≥ 15 y of age that had no high-school diploma, the employment/population ratio of people ≥ 15 y of age, and average income of residents ≥ 15 y of age living in the census enumeration area. This dimension assesses the ability of area residents to obtain the goods and services that are a part of everyday life. Social deprivation, on the other hand, assesses the strength of family social ties within an area. Factor scores were calculated from the indicators: proportion of persons ≥ 15 y of age that were separated, divorced or widowed, proportion of people ≥ 15 y of age that lived alone, and proportion of single-parent families who were living in the enumeration area. All indicators used (except for single-parent family) were standardized for the age structure of the Québec population before being included in the principal components analysis [54].

Factor scores for each dimension were categorized into quintiles, where increasing quintiles indicate increasing deprivation. To facilitate interpretability in longitudinal analysis, both were dichotomized into low (quintiles 1–3) and high deprivation (quintiles 4–5) as has been done elsewhere [55].

These variables could change over time, but in this analysis they were entered into models as time-invariant as they were not available in other cycles. This is due mainly because the Canadian census is conducted once every five years. An update, based on the 2001 census, linked to postal codes was not available for this study, but descriptive data showed that these measures remained largely unchanged from 1996–2001, and in fact, were becoming more homogeneous in their deprivation status [56]. Children could also have moved to a different enumeration area, but the ISQ did not update the deprivation indices each time a child moved.

### Neighborhood social cohesion and disorder

Social cohesion and disorder were based on scales derived from the work of Barnes-McGuire, which have been previously validated and used in the Canadian National Longitudinal Survey of Children and Youth [47]. Both variables assess the mother’s perceptions of her neighborhood and were collected every other data collection cycle, starting at five months of age (first data collection cycle). For the purposes of this analysis, only the responses at ages four, eight, and 10 y were required; meaning both were analyzed as time-dependent variables.

Five items with a Likert-type response scale form the social cohesion measure, a general measure of trust and support of neighbors. The disorder scale assesses neighbourhood quality by asking about four types of problems, including drug use and drug-dealing, presence of garbage and broken glass, public drinking, and groups of young people causing trouble. Response categories for this scale consist of: “a problem”, “somewhat of a problem”, and “not a problem”. Scale scores for each were calculated by averaging the item scores. The social cohesion score ranges from 1 to 4 with higher scores indicating a less cohesive neighbourhood. Disorder ranges from 1 to 3 with lower scores indicating more problems. Both scales have demonstrated adequate internal consistency with each originally calculated to have a Cronbach’s alpha of ≥ 0.75 [57].

In order to increase interpretability, both scales were dichotomized. For social cohesion, this was based on the 50^th^ percentile, as has been done by other studies using similar scales [34]. Following the methods of Curtis et al. (2004), disorder was dichotomized into: “any problems present” versus “no problems present” [58], as its distribution was highly skewed.

### Living location

Postal codes were recorded in all data collection cycles and linked to Statistics Canada’s census geographical areas by the ISQ. In this study, household locations were classified into one of three different types of areas: 1) Census Metropolitan Area (CMA) with ≥ 100,000 inhabitants; 2) Census Agglomeration (CA) with 10,000 to < 100,000 inhabitants; or 3) rural/small town with < 10,000 inhabitants [59]. For methodological reasons, a living location variable was not derived when children were four y of age. In order to use this measure as a time-dependent variable, the value in the previous data collection cycle (at 3.5 y) was carried forward to four y, and the values at eight and 10 y were used in the analysis.

### Other explanatory variables

In order to more clearly understand the relationship between place factors and food insecurity, a number of potential confounders and other pertinent explanatory variables were identified from the literature [16,60] and were included in the multivariable model. Time-dependent variables measured at age four, eight, and 10 y included: SES (a composite measure of income, job status, and education of both parents that was derived by the ISQ); number of people living in the household; and single-parent family status. The theory and method behind derivation of the SES composite is described elsewhere [61]. Time-stable variables measured at baseline (four y) included: at least one person in the household smokes, self-reported immigrant status, and age of the mother. Sex of the respondent was not added as this was the mother in most cases (98%).

### Statistical analysis

SAS version 9.2 was used to conduct all analyses. Two-tailed significance tests with an alpha of 0.05 were used throughout. Initial exploratory analyses were carried out by graphically displaying the distributions of all considered variables, computing measures of central tendency and dispersion, and checking for outliers. Crude tests of association (chi-squared, ANOVA) were conducted to examine unadjusted relationships between food insecurity and the explanatory variables at baseline, when children were four y of age.

Of the original sample (n =2120), approximately 46% (n = 978) had all three food insecurity measures, almost 18% (n = 377) had two, and 27% (n =572) had one; 9% (n = 193) did not have any and therefore could not be included in the analysis. A further 181 were excluded due to the cumulative effect of missing explanatory variables. There was no association between food insecurity at four, eight, and 10 y and being excluded for having missing explanatory data (χ^2^, *P* > 0.05). Children with no food insecurity responses (n=193) were more likely to be disadvantaged (e.g. live in materially or socially deprived areas, have immigrant mothers, live in a household with a low SES, or a single parent) than those with at least one response (χ^2^, *P* ≤0.05).

To determine if changes in the place factors were associated with changes in food insecurity, a longitudinal logistic regression analysis was carried out using Generalized Estimating Equations (GEE), with robust standard errors calculated for the estimated regression coefficients. Time was modeled as a categorical predictor with age four y specified as the reference category. An unstructured working correlation matrix was initially specified to account for the correlations among repeated measures over time.

As a first step in model building, crude (unadjusted) associations between repeated measures of food insecurity and each considered explanatory variable were estimated using GEE models that included main effects for time, the explanatory variable (over time if time-dependent), as well as an interaction term for the explanatory variable with time. This was done to determine the explanatory variables’ unadjusted relationship with food insecurity over time.

A multivariable model was then estimated by including the main effects of time, and all considered explanatory variables together with their interactions with time. The model was reduced by removing non-significant time interaction terms via backwards elimination, with the level of significance set at 0.05. For this model, four different correlation structures were compared using the quasilikelihood (QIC) statistic [62] (unstructured, AR(1), toeplitz, and exchangeable). Given that respondents could be included even if they were missing some time-dependent data, sample weights were not used.

## Results

A total of n=1746 children were included in the analysis (82% of the original sample). Of these children, the prevalence of food insecurity was 9.2% (n= 158/1726, 95%CI: 7.8 – 10.6%) at four y of age. This decreased to 7.6% (n = 89/1169, 95%CI: 6.2 – 9.3%) at age eight, and to 7.1% (n = 72/1018, 95%CI: 5.6 – 8.8%) at age 10 ^a^ . Of food insecure families with at least two response points (n = 134), 57% experienced food insecurity once, while 43% experienced food insecurity two or three times (from age four to 10 y). Table 1 details crude associations between food insecurity and the place factors, as well as with the other explanatory variables, when the child was four y of age. All variables except living location, number of people living in the household, and mother’s age were significantly related to food insecurity at baseline. These were still included in the adjusted analysis as potential confounders.

**Table 1:** **Characteristics of households with children participating in the QLSCD and included in the analysis at age 4 y (2002), by food insecurity status**^**a b**^

	**Food insecurity status at 4 y**			
**Variables**	**Food secure 90.8% (n=1568)**	**Food insecure 9.2% (n =158)**	**Total % (n)**	**Total N**
**Place factors**				
Materially deprived	36.3 (569)	53.2 (84)	37.8 (653)	1726
Socially deprived	34.6 (542)	52.5 (83)	36.2 (625)	1726
Low social cohesion ^c^	50.5 (771)	69.1 (105)	52.2 (876)	1679
High social disorder ^c^	23.9 (374)	45.5 (71)	25.9 (445)	1719
Living location ^c^				1710
Rural/small town	22.1 (343)	22.6 (35)	22.1 (378)	
CA (semi-urban)	11.3 (176)	11.0 (17)	11.3 (193)	
CMA (urban)	66.6 (1036)	66.5 (103)	66.6 (1139)	
**Other explanatory variables**				
Number of people in household *(mean, SD)*^c^	4.1 (0.96)	4.3 (1.2)	4.1 (1.0)	1726
SES (tertiles) ^c^				1715
Low	28.8 (449)	62.8 (98)	32.0 (547)	
Medium	34.3 (535)	28.9 (45)	33.8 (580	
High	36.9 (575)	8.3 (13)	34.3 (588)	
Single parent family ^c^	11.0 (173)	26.0 (41)	12.4 (214)	1726
At least one person in household smokes	28.9 (453)	42.4 (67)	30.1 (520)	1726
Mother is an immigrant	8.1 (127)	17.7 (28)	9.0 (155)	1726
Age of the mother *(mean y, SD*)	33.3 (5.0)	32.5 (6.3)	33.2 (5.2)	1726

^a^ All variables except living location, number of people in the household, and age of the mother were significantly associated with food insecurity at the 5% level.

^b^ All variables (except where indicated in italics) are percentages (n).

^c^ Measured at 4 y (2002) here but used as a time-dependent variable in multivariate analysis.

QLSCD – Québec Longitudinal Study of Child Development; CA – census agglomeration; CMA – census metropolitan area; SD – standard deviation; SES – socioeconomic status.

In the multivariable model (Table 2), comparison of different correlation structures did not show any differences in the QIC. Given the large sample size and small number of repeated measures, the unstructured correlation matrix was preferred and all estimates reported are based on this correlation structure. The odds ratios for time suggested that as children aged, food insecurity became less likely in the household; however these associations were not statistically significant. There were no significant interactions between the explanatory variables and time in the crude or multivariable models. Therefore, odds ratios estimate the average association between the explanatory variable and food insecurity, while also controlling for time and correlations between the repeated measures of food insecurity. Among the place factors, social deprivation, low social cohesion, and high disorder significantly increased the odds of food insecurity, in the range of 45 – 76%, and this was independent of other explanatory factors. Material deprivation became non-significant when other explanatory variables were added to the model. There continued to be no significant association between living location and food insecurity in the multivariable model. Effect sizes for household-level factors such as SES and single-parent family status were much higher than the place factors. For instance, living in a low SES household was associated with an 8.5 fold increased odds of food insecurity (95% CI: 5.05 – 14.1) compared to living in a high SES household. One other explanatory variable was also significantly related to food insecurity: number of people in the household became significant in multivariable analysis, although with a relatively weak odds ratio (Table 2).

**Table 2:** GEE multivariable logistic regression model to measure the adjusted association between place and food insecurity among households with children participating in the QLSCD, 2002 – 2008 (n = 1746)

**Variables included in the model**	**Adjusted odds ratio**	**95% Confidence limits**
Time 1 (child 4 y, 2002)	Ref	Ref
Time 2 (child 8 y, 2006)	0.83	0.64 – 1.08
Time 3 (child 10 y, 2008)	0.77	0.58 – 1.02
**Place factors**		
Materially deprived	1.12	0.81 – 1.54
Socially deprived	1.62	1.16 – 2.26 **
Low social cohesion	1.45	1.10 -1.92 **
High disorder	1.76	1.37 – 2.27***
Living location		
Rural/small town	0.88	0.59 – 1.31
CA (semi-urban)	0.93	0.59 -1.46
CMA (urban)	Ref	Ref
**Other explanatory variables**		
Number of people in household	1.26	1.07 – 1.48**
SES (tertiles)		
Low	8.45	5.05 – 14.12***
Medium	3.51	2.10 – 5.86***
High	Ref	Ref
Single parent family	2.49	1.69 – 3.67***
At least one person smokes in the household	1.17	0.86 – 1.58
Mother is an immigrant	1.37	0.82 – 2.29
Age of the mother	1.00	0.97 – 1.03

* *P* ≤ 0.05, ** *P* ≤ 0.01, *** *P* ≤ 0.0001.

GEE – generalized estimating equations; QLSCD – Québec Longitudinal Study of Child Development; Ref – reference group; CA – census agglomeration; CMA – census metropolitan area; SES – socioeconomic status.

## Discussion

The present study represents a step forward in this area of research in that it considers families with young children and was conducted using a more robust (longitudinal) design compared to previous studies. Household SES was by far the most important predictor of food insecurity in this sample of Québec families; however even after controlling for household SES and other important variables, social deprivation, low social cohesion, and high disorder were related to an increase in the odds of food insecurity.

In this study, living in socially deprived areas significantly increased the odds of being food insecure. This relationship has not been extensively studied in the literature, but past research in Québec has shown that the prevalence of food insecurity is higher when both forms of deprivation, material and social, are found together [63]. Higher social deprivation may indicate less intra-household social interaction occurring in particular areas. Fewer immediate kinship ties may reduce the interconnectedness of informal and formal social networks that could provide material resources, such as food, and non-material supports such as information on local food programs.

Low neighborhood social cohesion was found to relate to increased odds of food insecurity. Low cohesion may indicate less trust and reciprocity among neighbours within the neighbourhood, less interaction and support from neighbours, and a reduced capacity to mobilize for collective action to address food security-related issues. Brisson & Altschul (2011) examined social cohesion in a low income population from 10 cities in the US, and found that it was inversely related to food insecurity, as measured by the question “In the last 12 months…was your family ever without enough money to buy food?” [33]. This same association held when individual responses were aggregated by neighbourhood. In contrast to the findings here, a study of elderly people living in New York City, did not find that neighbourhood social cohesion was significantly related to food insecurity [35]. This sample, however, was demographically much different from the one analyzed in this study.

Other studies have examined collective efficacy or neighbourhood social capital more generally; some with significant findings in the hypothesized direction [30-32], while others have had null results [34,36,37]. Overall, mixed results may have to do with different population demographics, varying definitions for predictor and outcome, or reliance on the cross-sectional study design.

Neighbourhood aesthetics or quality, as measured by disorder in this study, could lead residents to perceive the area as unsafe, and therefore avoid accessing services outside of the household. Perceptions by businesses and institutions that have the ability to invest in the area with respect to services and infrastructure relevant to food security may lead to decreased investment [27]. Thus, high disorder may also be a proxy for fewer services and/or weakened linkages to these services. No studies to our knowledge have examined the relationship between this specific construct and food insecurity.

Material deprivation was not associated with food insecurity in the multivariable analysis. This may be because the other place factors mediated the association between material deprivation and food insecurity. Although they did not analyze food insecurity as an outcome, Sampson and Raudenbush (1997) determined that collective efficacy, comprised of social cohesion and informal social control, mediated the effect of neighbourhood disadvantage on violence in their study of 343 Chicago neighbourhoods [64]. One study that used an adapted version of the 6-item USDA FSSM, found that higher material deprivation was related to food insecurity in a clinic-based cohort of women with young children living in the UK [65]. Another study, conducted on a representative sample of Southern Australians, did not find that material deprivation was related to food insecurity as measured using the food depletion item of the Radimer/Cornell instrument [37]. Similar to our study, the Australian study included measures of neighbourhood social capital in the model, whereas, the UK study did not.

Living location was not found to be important in this study. This is in contrast to the literature, which has shown a fairly consistent association between rurality and decreased odds of food insecurity [19,36,38-42]. These studies were based for the most part on US samples and used the 18- or 6-item versions of the USDA FSSM. Differences with the present study may also reflect different contexts. Many health and social programs and services are provided free to residents of Québec, which may prevent food insecurity irrespective of broad geographical location. A network of local community service centers (CLSCs) covering the entire province provides many of these services [66]. Each one is mandated to respond to the needs of the local area, and targets all social classes [66]. This, therefore, may help to reduce geographical inequalities in access to food. These findings demonstrate the limitations of generalizing some place-related factors between culturally, politically, or geographically distinct jurisdictions.

The results of the present study should be interpreted in light of some important limitations. First, sample weights were not used in this analysis, so the proportion of families estimated to be food insecure is not necessarily generalizable to the entire population of children born in Québec in 1997–1998. The definition of food insecurity was not based on the full USDA FSSM [67] and now used by Health Canada [68]. Therefore, it cannot estimate the prevalence of food insecurity comparable to the FSSM as it does not capture all dimensions, but it does provide an overview of important food insecurity components [50]. Additionally, the third question relating to providing balanced meals may be interpreted differently in non-English speaking populations [69]. However, this three question measure has been developed from a study of a Québec, French-speaking population using the entire 13-item measure, and is used in other large government surveys in Québec [50]. Additionally, a proportion of households in the QLSCD were native English speakers, and received their questionnaires in English.

Another limitation of this study is that the analysis did not control for car ownership or investigate effect modification of car ownership with the place factors considered. The significant relationships seen in the present study may exist only for those households that do not have a car; they may be less strong or non-existent for those that do have a car [70]. Car ownership, on the other hand, is related to income level, where lower income households would be less likely to own a car. Overall SES was controlled for in the present analysis.

Although the longitudinal study design is methodologically more robust than cross-sectional studies reported in the literature, the present study is limited by problems of attrition and non-response. Nonetheless, households excluded because they had no food insecurity responses were more likely to be disadvantaged, as measured by many different variables including SES, single-parent status, immigrant status, and area material and social deprivation. Because food insecurity is so tightly linked to disadvantage, one would expect that these non-responders would be more likely to be food insecure at some point than the average participating household in the cohort, which should then lead to similar or stronger relationships as those uncovered among included households.

Finally, data on neighborhood material and social deprivation were only available when children were five months of age. It is unknown if families moved to areas higher or lower in either of the two forms of deprivation. However, in general, families living in highly deprived areas tend to move laterally, to other deprived areas, rather than move to more affluent areas [71]. Canadian data indicate that new movers to deprived areas tend to stay on average for four years before moving out, and that the longer one stays in a deprived neighbourhood, the less likely they are to leave [72]. Additionally, American data show that it is common for families to return to high poverty areas after leaving for a period of time [73].

## Conclusion

From this study it is likely that improving the household SES situation (e.g. increase household income), would lead to decreased food insecurity. But our results suggest that addressing the immediate social environment in which people live may also be of benefit. More studies are needed to verify these results, and to delve more deeply into specific features of the physical and social characteristics of local environments. It is also pertinent to understand how these environmental factors might interact with household SES and other household factors that affect food utilization. These social environmental factors may also explain why communities may be food secure but not the individuals residing within them. Thus, various stakeholders (e.g. urban planners, politicians, dietitians, social workers, health promoters) may want to consider local social environments when implementing any type of food security intervention in the community. Future research may show that the environment itself may be amenable to change by certain interventions, which could lead to improved food security and health-related outcomes. Consideration for interactions with the environment and changes to the environment itself may lead to sustainable food system reforms and thus improve both individual/household and community food security. Some examples include community capacity building and empowerment initiatives to build social capital, designing the built environment to encourage social interaction and prevent anti-social behaviour, and providing more targeted social support to isolated households. Planning and evaluating these interventions with individual and community food insecurity as outcomes should become a priority.

## Endnote

^a^Denominators differ as not all children responded in each data collection cycle, but because of the longitudinal regression method used, these children are still included in the analysis.

## Abbreviations

CA: Census agglomeration; CMA: Census metropolitan area; FSSM: Food Security Survey Module; GEE: Generalized estimating equations; ISQ: Institut de la statistique du Québec; QLSCD: Québec Longitudinal Study of Child Development; Ref: Reference group; SD: Standard deviation; SES: Socioeconomic status; USDA: United States Department of Agriculture.

## Competing interests

The authors declare that they have no competing interests.

## Authors’ contributions

MAC conceived of the study, conducted the analysis, and drafted and edited the manuscript; LD helped to conceive the study, provided content expertise, and critically edited all versions of the manuscript; MST provided content expertise and critically edited all versions of the manuscript; MT provided statistical expertise and critically edited all versions of the manuscript. All authors have given approval for the final version of this manuscript to be published.

## Disclaimer

This analysis was based on the ISQ QLSCD master files. All computations were prepared by MAC. The responsibility for the use and interpretation of these data is solely that of the authors. The opinions expressed in this paper are those of the authors and do not represent the views of the ISQ.

## Pre-publication history

The pre-publication history for this paper can be accessed here:

http://www.biomedcentral.com/1471-2458/12/1038/prepub
